# The Regulation of Lipid Deposition by Insulin in Goose Liver Cells Is Mediated by the PI3K-AKT-mTOR Signaling Pathway

**DOI:** 10.1371/journal.pone.0098759

**Published:** 2015-05-06

**Authors:** Chunchun Han, Shouhai Wei, Fang He, Dandan Liu, Huofu Wan, Hehe Liu, Liang Li, Hongyong Xu, Xiaohui Du, Feng Xu

**Affiliations:** Institute of Animal Breeding & Genetic, Sichuan Agricultural University, Chengdu, Sichuan 611130, P.R. China

## Abstract

**Background:**

We previously showed that the fatty liver formations observed in overfed geese are accompanied by the activation of the PI3K-Akt-mTOR pathway and an increase in plasma insulin concentrations. Recent studies have suggested a crucial role for the PI3K-Akt-mTOR pathway in regulating lipid metabolism; therefore, we hypothesized that insulin affects goose hepatocellular lipid metabolism through the PI3K-Akt-mTOR signaling pathway.

**Methods:**

Goose primary hepatocytes were isolated and treated with serum-free media supplemented with PI3K-Akt-mTOR pathway inhibitors (LY294002, rapamycin, and NVP-BEZ235, respectively) and 50 or 150 nmol/L insulin.

**Results:**

Insulin induced strong effects on lipid accumulation as well as the mRNA and protein levels of genes involved in lipogenesis, fatty acid oxidation, and VLDL-TG assembly and secretion in primary goose hepatocytes. The stimulatory effect of insulin on lipogenesis was significantly decreased by treatment with PI3K-Akt-mTOR inhibitors. These inhibitors also rescued the insulin-induced down-regulation of fatty acid oxidation and VLDL-TG assembly and secretion.

**Conclusion:**

These findings suggest that the stimulatory effect of insulin on lipid deposition is mediated by PI3K-Akt-mTOR regulation of lipogenesis, fatty acid oxidation, and VLDL-TG assembly and secretion in goose hepatocytes.

## Introduction

Insulin plays a major role in the regulation of carbohydrate and lipid metabolism in the liver, adipose tissue, and muscle. Hepatic fatty acid oxidation, lipogenesis, and protein synthesis are subject to regulation by insulin [1]. More specifically, insulin controls the synthesis of lipids from glucose in the liver and adipose tissue and controls the export of fatty acids (FAs) and lipoproteins from the liver to extrahepatic organs. A relationship between lipid deposition and activation of the PI3K-Akt-mTOR (phosphatidylinositol 3-kinase-protein kinase B-mammalian target of rapamycin) pathway has been confirmed in hepatitis patients [2,3]. PI3 kinases comprise a family of related intracellular signal transducer enzymes that can phosphorylate the 3 position hydroxyl group of the inositol ring of phosphatidylinositol. This phosphorylation event results in the activation of protein kinase B, also known as Akt. PI3K is thus linked to the extraordinarily diverse array of cellular functions regulated by downstream components of this pathway, including cell growth, proliferation, differentiation, and motility [4]. Recently, Jackel-Cram et al. revealed that hepatitis C virus genotype3a core protein cause liver steatos is through activation of the PI3K-Akt pathway, indicating that the activated PI3K-Akt pathway functions in lipogenesis [2]. PI3K has been shown to mediate insulin stimulation of the promoter of fatty acid synthase (FAS), a critical enzyme involved in lipogenesis [5]. However, the definitive molecular mechanisms by which the PI3K-Akt-mTOR pathway participates in insulin-induced lipid deposition have not been fully elucidated.

In avian species, lipogenesis takes place primarily in the liver, which accounts for 95% of de novo FA synthesis. It has been reported that overfeeding geese with a carbohydrate-rich diet results in a dramatic increase in hepatic lipid deposition and the induction of liver steatosis [6,7]. We have found that overfeeding geese clearly alters plasma insulin concentrations as well as the protein content and mRNA levels of genes involved in the PI3K-Akt-mTOR pathway. To verify the role of the PI3K-Akt-mTOR pathway in insulin-induced lipid deposition, we investigated whether inhibition of PI3K-Akt-mTOR signaling in goose primary hepatocytes would affect insulin-induced alterations in major lipid metabolic pathways.

## Materials and Methods

### Ethics Statement

All animal studies were approved by the Animal Care and Use Committee of Sichuan Agricultural University.

### Primary Hepatocyte Isolation and Culture

Hepatocytes were isolated from three 30-day-old Sichuan White geese from the Experimental Farm for Waterfowl Breeding at Sichuan Agricultural University using a modified version of the two-step procedure described by Seglen [8]. This method differed from that of Seglen in that the liver was removed before the preperfusion step. The geese were cleared with disinfectant, and heparin sodium (100 IU/kg body weight) was used by intravenous injection. And then anesthesia was induced by intraperitoneal injection with 3% isoflurane (35mg/kg body weight). After the geese fell into a coma, the abdominal cavity was slited open along the median line of abdomen, and the liver was taken out rapidly and cleaned with 37°C physiological salt solution. Immediately, the jugular vein was cut and geese were bled. Then the following procedure was the same with the two-step procedure described by Seglen [8]. Cell viability was greater than 90%, as assessed by the trypan blue dye exclusion test. Freshly isolated hepatocytes were diluted to a concentration of 1×10^6^ cells/ml. The culture medium was composed of DMEM (containing 4.5 g/L glucose; Gibco, USA) supplemented with 100 IU/ml penicillin (Sigma, USA), 100 μg/ml streptomycin (Sigma, USA), 2 mM glutamine (Sigma, USA), and 100 ml/L fetal bovine serum (Clark, Australia). The hepatocytes were either plated in 60-mm culture dishes at a density of 3×10^6^ cells per dish for total RNA and nuclear protein isolation or in 24-well plates at a density of 1×10^6^ cells per well to measure the triglyceride (TG) levels and very low density lipoprotein (VLDL) concentrations. The cultures were incubated at 40°C in a humidified atmosphere containing 5% CO_2_. The media was refreshed after 3 h; after 24 h, the media was replaced with serum-free media (insulin was not added to the serum-free media). After an additional 24 h, the cells were treated with serum-free media supplemented with 0, 50, 100, or 150 μmol/L insulin and incubated for 24 h without refreshing the media. Control cells were cultured with serum-free media for 24 h without refreshing the media. Additionally, some cells were treated with serum-free media supplemented with PI3K-Akt-mTOR pathway inhibitors (LY294002, rapamycin, and NVP-BEZ235, respectively) for 24 h, followed by the addition of 50 or 150 nmol/L insulin and incubation for an additional 24 h. At the end of the incubation period, the culture media and cells were cooled on ice and collected. All experiments were repeated three times.

### Measurement of Intracellular and Extracellular TG Concentrations

The culture media was collected to determine the concentration of extracellular TG. The cell samples used to measure intracellular TG concentrations were collected and shaken for 1 h using an ultrasonic processor and then directly added to 0.5 ml of an isovolumic mixture of chloroform and methanol (2:1, v/v). TG levels were quantified using a colorimetric method [9] and a triglyceride GPO-POD assay kit (Biosinc, China).

### Measurement of Extracellular VLDL Concentration

The media samples used to measure extracellular VLDL concentrations were collected and centrifuged for 20 min at 1000 g. The VLDL concentration of the supernatant was measured using a chicken VLDL ELISA kit (GBD, USA) according to the manufacturer’s instructions. The microtiter plate provided with the kit was pre-coated with an antibody specific to VLDL. The enzyme-substrate reaction was terminated by the addition of a sulfuric acid solution, and the color change was measured spectrophotometrically at a wavelength of 450 nm. The concentration of VLDL in the samples was determined by comparing the optical density (OD) values of the samples to a standard curve.

### Measurement of Protein Contents in Cultured Cells

The protein levels of ACCα, FAS, and CPT1in the cultured cells were measured using the appropriate ELISA kits according to the manufacturer’s instructions (MyBioSource, Inc., USA). First, 50 μl of standard or sample was added to the appropriate well of the antibody pre-coated plate, then 10 μl of the biological reagent was added to the sample wells (but not the standard wells). Next, 100 μl of the enzyme conjugate was added to each well, and the plate was incubated for 1 h at room temperature. The plate was then washed five times, and after completely removing excess solution by tapping on absorbent paper, 100 μl of substrates A and B were added to each well. After 15 min of incubation at room temperature, 100 μl of stop solution was added, and the absorbance at 450 nm was read using a plate reader. The protein content in the samples was calculated from the polynomial second order or exponential standard curves obtained from the standards included in each assay.

### Oil Red O Staining

After the treatment with insulin and signal pathway inhibitors, the hepatocytes were stained with Oil Red O to assess the amount of fat accumulation. The hepatocytes (4×10^4^ cells/well) were cultured on four-well culture slides, fixed in formalin, and stained using a previously described method [10]. Briefly, the wells were fixed with Baker’s formalin for 15 min, rinsed with distilled water, equilibrated in 100% propylene glycol for 2 min, and stained with Oil Red O for 10 min. The wells were then treated with 60% propylene glycol (v/v) for 1 min to remove the free Oil Red O and rinsed with distilled water. The Oil Red O was extracted by the addition of isopropanol, and the Oil Red O content was determined in aliquots from the wells after shaking the culture plates for 30 min at room temperature. The cells were examined by phase contrast microscopy at 200× magnification.

### Oil Red O Extraction

The steps for Oil Red O extraction were similar to the protocol described above for Oil Red O staining with a previously described method [11]. The cells were washed three times with PBS and fixed with 10% formaldehyde for 30 min at room temperature. After being washed with PBS three times, the cells were stained with 1% filtered Oil Red O for 40 min at room temperature. The Oil Red O solution was then removed, and the cells were not washed. The intracellular triglycerides in the cells were extracted via agitation with 2000 μl of 100% isopropanol for 15 min in a shaker. Finally, a well containing DMSO was used to adjust the zero level, and the OD value of each well was monitored by a spectrophotometer at 510 nm.

### Isolation of Total RNA and Real-Time RT-PCR

Total RNA was isolated from cultured cells using TRIzol (Invitrogen, USA) and reverse-transcribed using the Primer Script TM RT system kit for real-time PCR (TaKaRa, Japan) according to the manufacturer’s instructions. The quantitative real-time PCR reactions contained the newly generated cDNA template, SYBR Premix Ex Taq TM, sterile water, and primers for the target genes. Real-time PCR was performed on a Cycler system with one cycle of 95°C for 10 s, followed by 40 cycles of 95°C for 5 s and 60°C for 40 s. An 80-cycle melt curve was performed to determine the primer specificity, starting at 55°C and increasing by 0.5°C every 10 s. Specific primers were designed according to goose gene sequences and are listed in Table 1.

**Table 1:** Primer sequences for real-time PCR.

Gene name	Upstream (5'-3')	Downstream (5'-3')	Product size (bp)	Accession number
SREBP-1	CGAGTACATCCGCTTCCTGC	TGAGGGACTTGCTCTTCTGC	92	EU333990
FAS	TGGGAGTAACACTGATGGC	TCCAGGCTTGATACCACA	109	EU770327
ACCα	TGCCTCCGAGAACCCTAA	AAGACCACTGCCACTCCA	163	EF990142
PPARα	ATCTATCCCTGGCTTCTCCA	AGCATCCCATCCTTGTTCATT	117	AF481797
MTTP	CCCGATGAAGGAGAGGAA	AAAATGTAACTGGCCTGAGT	85	GO240734
FoxO1	CATCCCTTCAGTCTGGTCAA	GAAAGGCTGGGTAAAGTAG	265	GW342986
CPT1	GTCTCCAAGGCTCCGACAA	GAAGACCCGAATGAAAGTA	193	GW342945
ACOX1	ACAGAAAGAGCAAGGAGGAT	GCACGAGGTCAACAGAAGT	51	KC424582
ApoB	CTCAAGCCAACGAAGAAG	AAGCAAGTCAAGGCAAAA	153	GW342984
PPARγ	CCTCCTTCCCCACCCTATT	CTTGTCCCCACACACACGA	108	AF481798.1
LXRα	CCCAGCCCTTCCCACAAACT	CTGCCTCGCTTCACGGTTATTAG	156	HM138512
ChREBP	AAGAAGCGGCTCCGAAAG	TGGTGGGTGCTGGGTGT	236	GW342987.1
β-actin	CAACGAGCGGTTCAGGTGT	TGGAGTTGAAGGTGGTCTCG	92	M26111.1
18S	TTGGTGGAGCGATTTGTC	ATCTCGGGTGGCTGAACG	129	L21170.1

To confirm the presence of a unique band of the expected size, amplicons corresponding to each target were examined on an agarose gel. Negative controls, which consisted of PCR reactions with non-reverse transcribed RNA, did not generate any signal. All samples were amplified in duplicate with the same PCR mixture and in the same 96-well plate. The cycle threshold variation observed between the duplicates was on average 0.12 ± 0.1, demonstrating a high level of intra-assay reproducibility. Each sample was also replicated in another 96-well plate, and the Ct variation between the two independent plates was 0.28 ± 0.22, showing a fair level of inter-assay reproducibility. PCR products were then diluted 16-fold and used to generate a calibration curve and the amplification rate (R) for each gene. For each experimental sample, a normalized target gene expression level (Exp), corresponding to the target gene expression level relative to the β-actin, 18S, and UBS (housekeeping genes) expression levels, was determined using the 2^-ΔΔCt^ method as previously described [12]:

$${\text{Exp}}_{\text{target gene in sample}}={\left(1+{\text{R}}_{\text{target gene}}\right)}^{\text{Ct}\left(\text{target gene in sample}\right)}/{\left(1+{\text{R}}_{\beta -\text{actin or}18\text{S or UBS}}\right)}^{\text{Ct}\left(\beta -\text{actin or}18\text{S or UBS in sample}\right)}$$

For the target gene expression analysis, the normalized target gene expression level for each sample was compared to the positive control sample. The relative mRNA levels areexpressed as the n-fold difference in the normalized target gene expression level between each treated and control sample. The final values were calculated by extracting the square root of the two relative mRNA levels of each gene relative to β-actin, 18S and UBC. The assays for each individual gene were repeated three times and averaged.

### ACCα Protein Analysis by Western Blot

Hepatocytes were washed twice and collected in ice-cold PBS. Total protein extracts were obtained using a reducing SDS buffer containing 50 mmol/L Tris-HCl (pH 6.8), 100 mmol/L DTT, 2% SDS, and 10% glycerol. Protein concentrations were determined for diluted samples using the Bradford procedure. Equal amounts of protein (100 μg) were separated by 6% SDS-PAGE and transferred onto membranes. The membranes were blocked in a TBS solution containing 5% nonfat dry milk and incubated with an antibody against ACCα (1:1000 dilution; Beijing Biosynthesis Biotechnology, China). A goat anti-rabbit horseradish peroxidase-conjugated IgG (1:2000; Beijing Biosynthesis Biotechnology, China) was used as the secondary antibody, and the signals were detected using an ECL western blot detection kit (Beyotime Institute of Biotechnology, China). After analysis, the membranes were blotted with ananti-α-tubulin antibody (1:1000; Beijing Biosynthesis Biotechnology, China) to normalize for protein content. The blot images were digitized using a luminescent image analyzer (LAS-1000, Fuji Photo Film). The protein bands were quantified using Quantity One software (Bio-Rad). The relative ACCα protein level was determined based on the value of ACCα expression divided by the value of α-tubulin expression. Each individual experiment was repeated three times and averaged.

### Statistical Analysis

Data were statistically analyzed using one way ANOVA with Tukey HSDor Dunnett T3 multiple comparison test to isolate individual differences. Analyses were performed using GraphPad Prism software. * indicate statistical significance with p<0.05. Every experiment was repeated with three biological samples, and each sample was run in triplicate.

## Results

### Insulin Stimulates Lipid Deposition in Goose Hepatocytes

**Fig 1:**
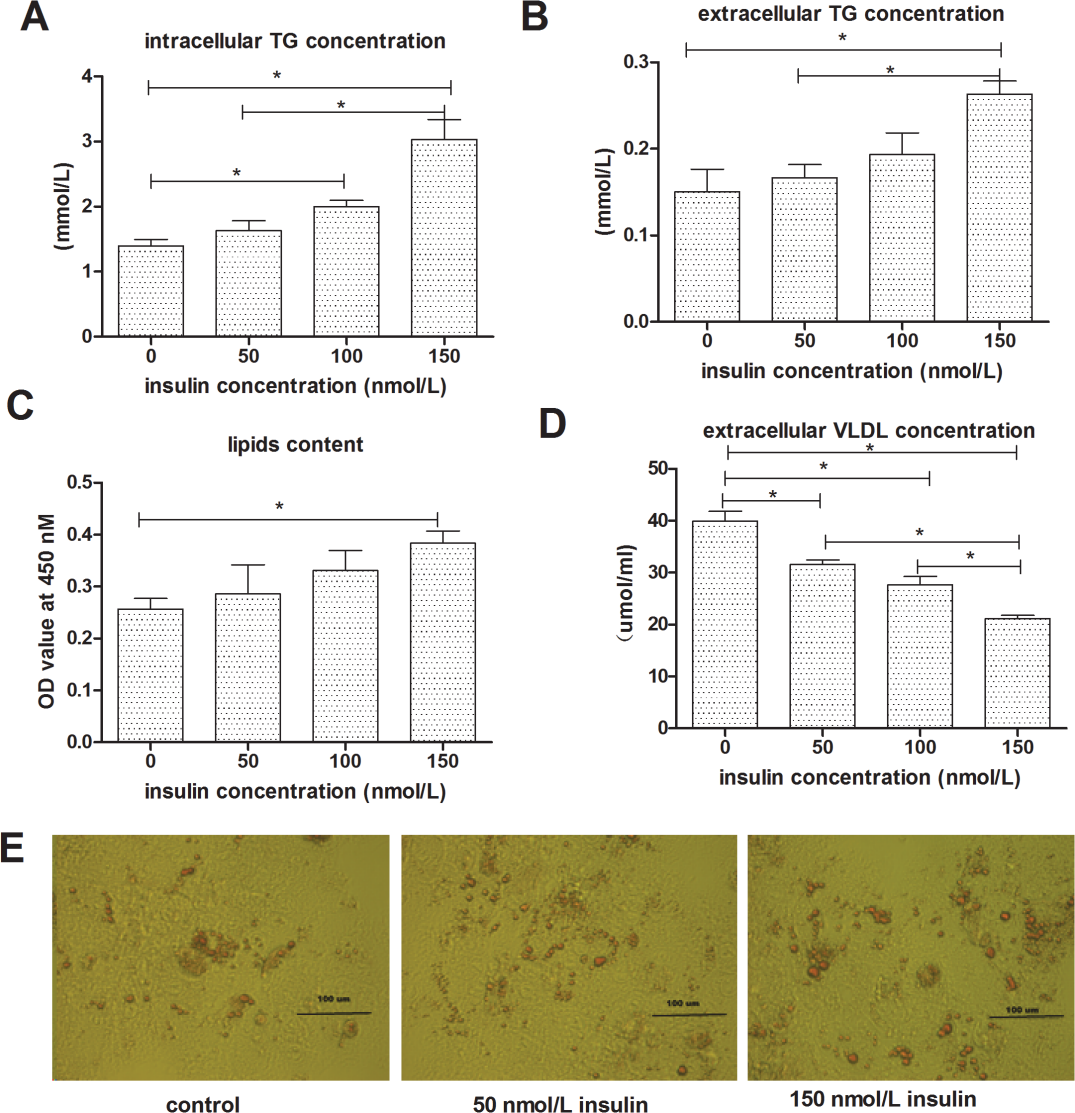
Effect of insulin on lipid deposition. **A**, intracellular TG concentrations. **B**, extracellular TG concentrations. **C**, extracellular VLDL concentrations. **D**, Lipid contents were measured by Oil Red O extraction and are shown in optical density value units. **E**, intracellular lipid accumulation measured by Oil Red O staining; the cells were examined by phase contrast microscopy at 40× magnification.

Compared with the control group, incubation with insulin increased the intracellular TG concentration, the extracellular TG concentration, (Fig 1A and 1B), and decreased the extracellular VLDL concentration also in a dose-dependent manner. The 150 nmol/L dose of insulin had the strongest effect on all three of these parameters.

To investigate the effects of insulin on fat deposition, we measured lipid accumulation in hepatic cells after exposure to different concentrations of insulin. Intracellular lipid vacuoles were visualized by phase-contrast microscopy and confirmed by Oil Red O staining. Whereas minimal lipid staining was observed in untreated cells, lipids accumulated in a dose-dependent manner following the addition of insulin. Fig 1E shows are presentative result in which goose primary hepatocytes treated for 24 h with different concentrations of insulin exhibited lipid droplet accumulation compared with control cells. This level of lipid droplet accumulation was observed in almost all of the insulin-treated cells. The lipid contents measured with the Oil Red O extraction assay (Fig 1D) were consistent with the Oil Red O staining patterns, and 150 nmol/L dose of insulin had an evident increase on the lipids content (p<0.05).

**Fig 2:**
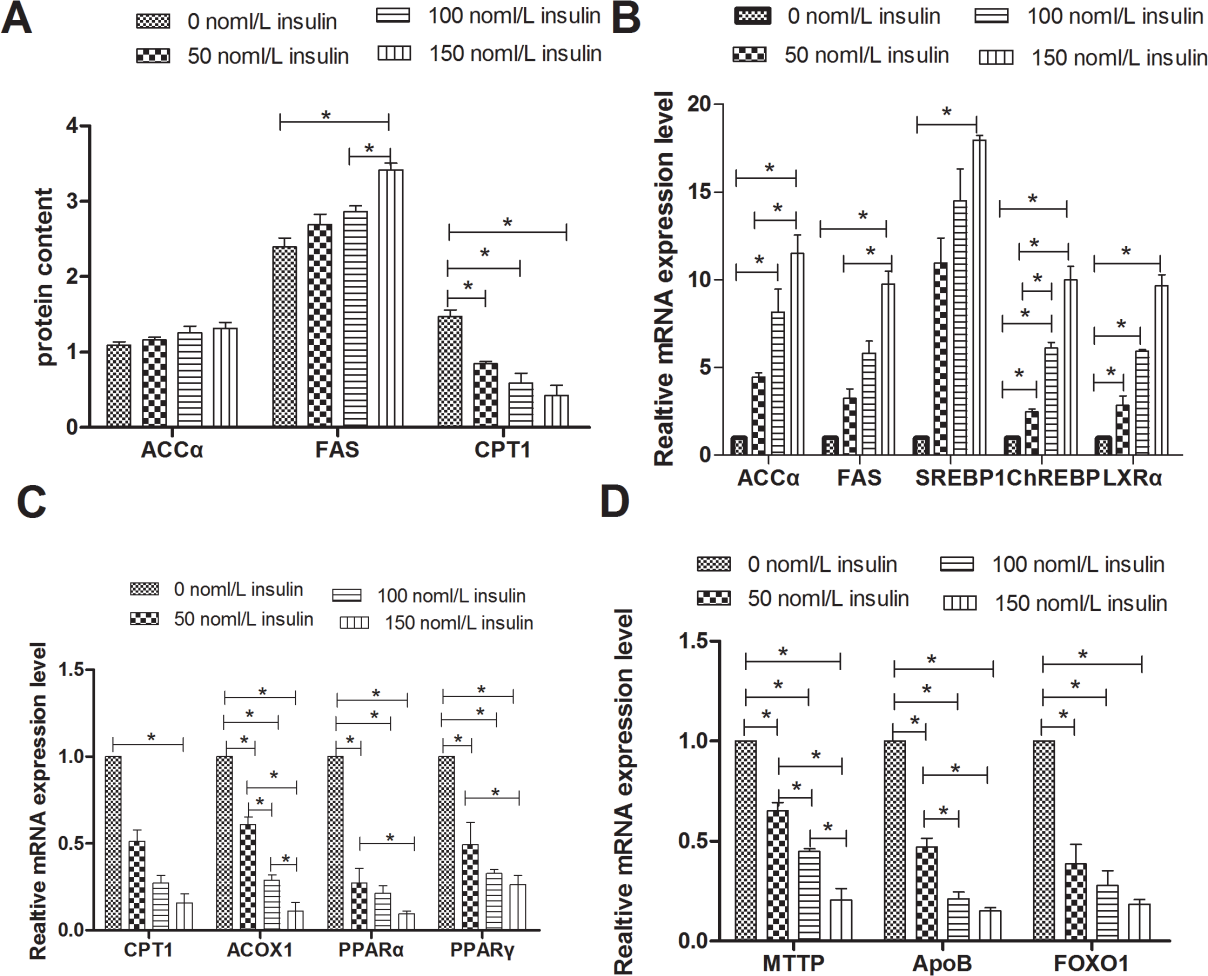
Effect of insulin on protein levels and mRNA levels of genes involved in lipid metabolism. **A**, Protein levels of genes involved in lipid metabolism; FAS levels are shown in nmol/ml, whereas ACCα and CPT1 levels are shown in ng/ml. **B**, Relative mRNA levels of genes related to lipogenesis. **C**, Relative mRNA levels of genes involved in fatty acid oxidation. **D**, Relative mRNA levels of genes that participate in VLDL-TG assembly and secretion. * indicate significant differences among treatments (P<0.05).

Fig 2A summarizes the effect of insulin on protein levels of acetyl-CoA carboxylase-α (ACCα), fatty acid synthase (FAS), and carnitine palmitoyl transferase 1 (CPT1). Treatment with 50, 100or 150 nmol/L insulin decreased CPT1 protein levels in a dose-dependent manner, whereas all three doses of insulin increased FAS and ACCα protein levels relative to controls, and only 150 nmol/L insulin had a significant effect on FAS protein level(p<0.05).

**Fig 3:**
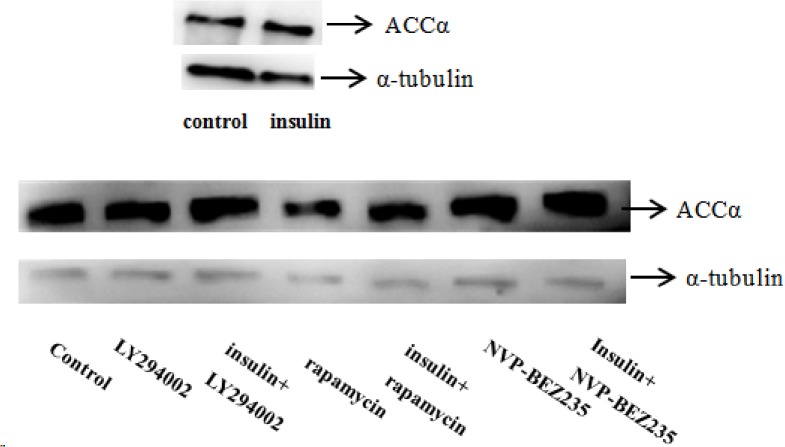
Treatment with LY294002, rapamycin, or NVP-BEZ235 decreased the insulin-stimulated up-regulation of ACCα protein expression. The concentrations of insulin, LY294002, rapamycin, and NVP-BEZ235 were 150 nmol/L insulin, 20 μmol/L, 30 nmol/L, and 1 μmol/L NVP-BEZ235 respectively. The blots are representative of three independent experiments.

Compared with the control, treatment of goose primary hepatocytes with 50, 100 or 150 nmol/L insulin dose-dependently increased the mRNA expression levels of genes involved in lipogenesis (SREBP-1, FAS, ACCα, ChREBP [carbohydrate response element binding protein], and LXRα) (Fig 2B). By contrast, 24 h of treatment with 50, 100 or 150 nmol/L insulin resulted in a dose-dependent down-regulation of genes involved in fatty acid oxidation (peroxisome proliferator activated receptor [PPARα], PPARγ, CPT1, acyl-CoA oxidase 1, and palmitoyl [ACOX1]) or VLDL-TG assembly and secretion (forkhead box O1 [FoxO1], microsomal triglyceride transfer protein [MTTP], and apolipoprotein B [ApoB]) (Fig 2C and 2D). A western blot assay confirmed that insulin up-regulated ACCα protein levels (Fig 3).

### LY294002 Reversed the Effect of Insulin on Lipid Accumulation

**Fig 4:**
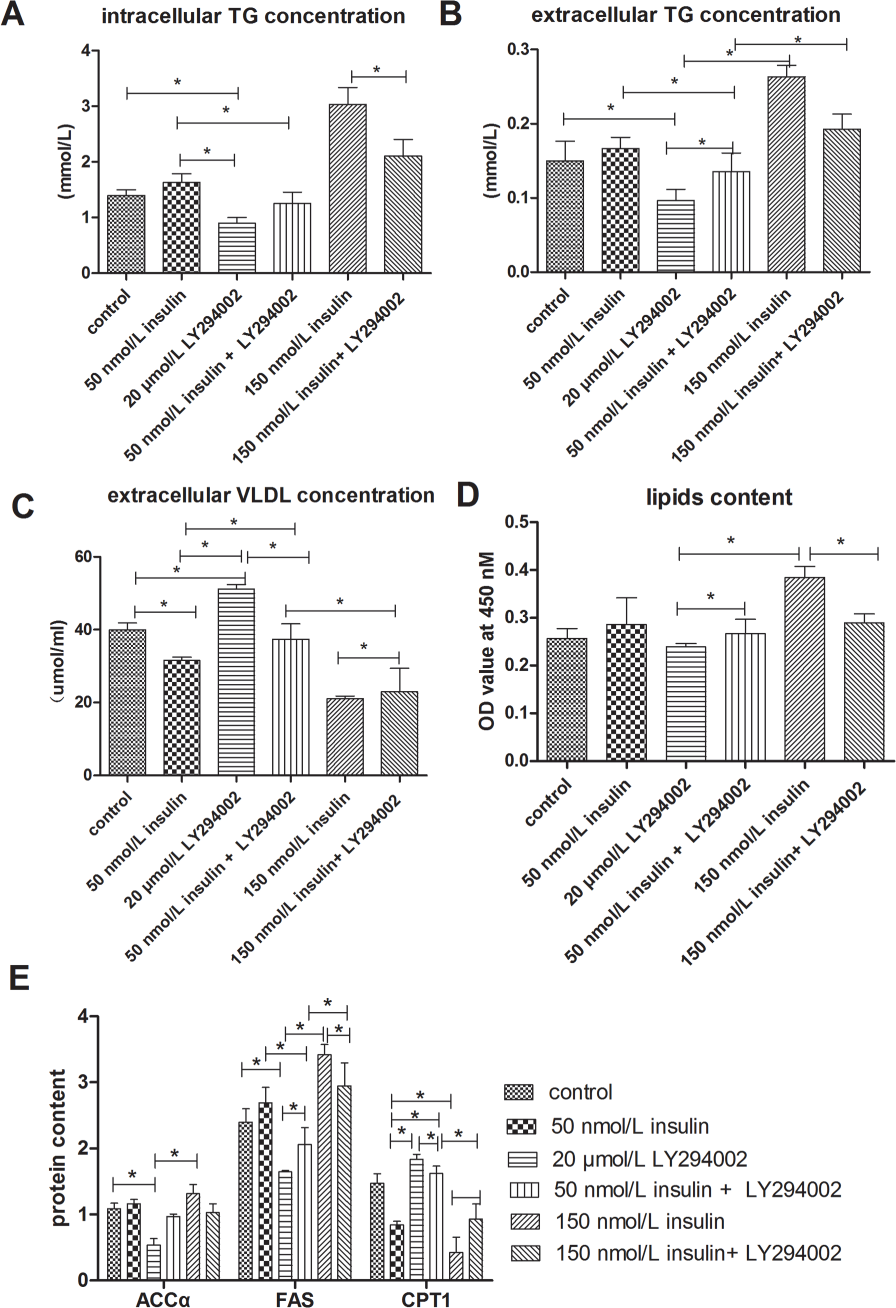
Treatment withLY294002 blocked the effect of insulin on lipid accumulation. **A**, intracellular TG concentrations. **B**, extracellular TG concentrations. **C**, extracellular VLDL concentrations. **D**, Lipid contents were measured by Oil Red O extraction and are shown in optical density value units. E, Protein levels of genes involved in lipid metabolism; FAS levels are shown in nmol/ml, whereas ACCα and CPT1 levels are shown in ng/ml. * indicate significant differences among treatments (P <0.05).

**Fig 5:**
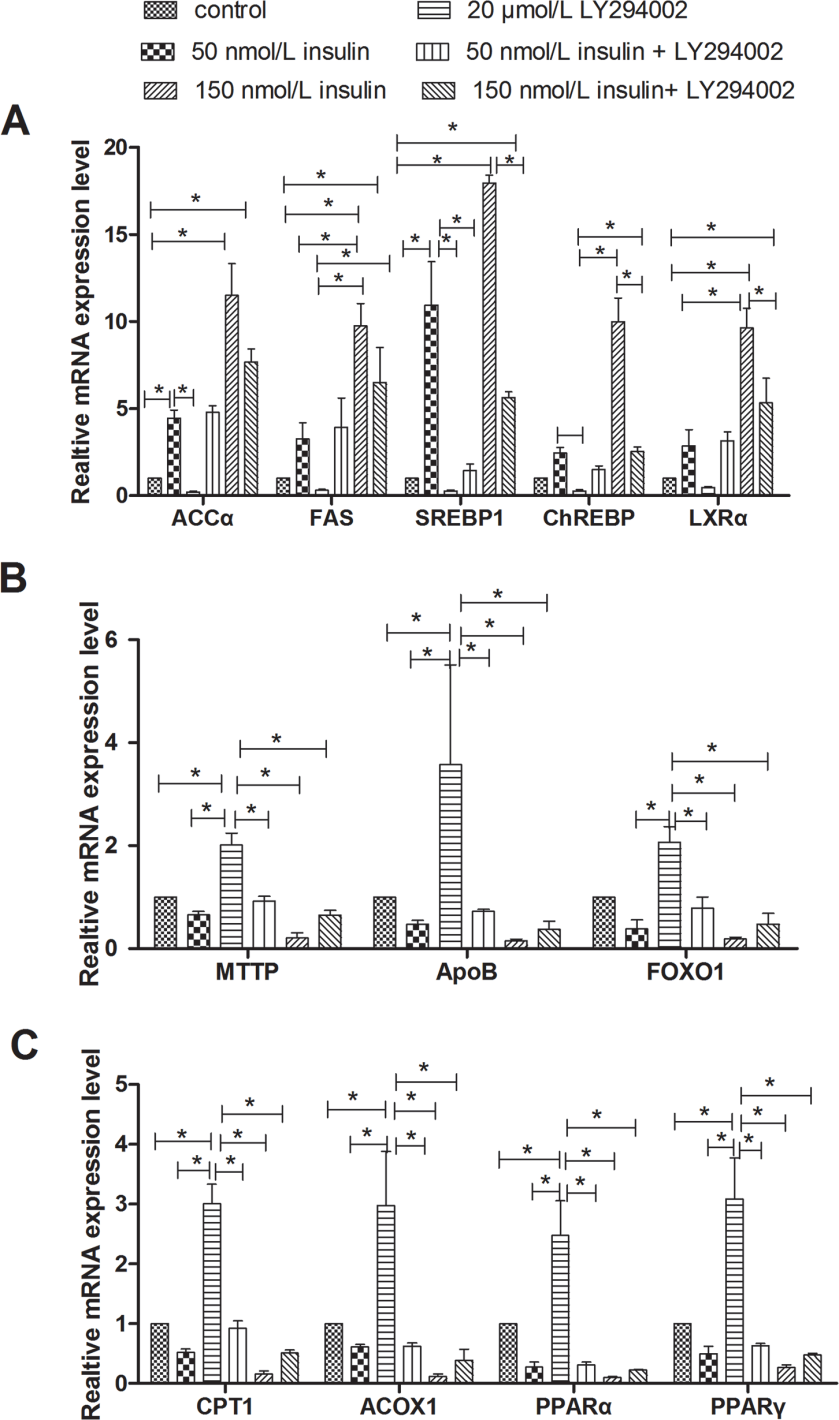
Treatment with LY294002 affected the regulation of insulin on mRNA levels of genes involved in lipid metabolism. **A**, Relative mRNA levels of genes related to lipogenesis. **B**, Relative mRNA levels of genes involved in fatty acid oxidation. **C**, Relative mRNA levels of genes that participate in VLDL-TG assembly and secretion.* indicate significant differences among treatments (P<0.05).

**Fig 6:**
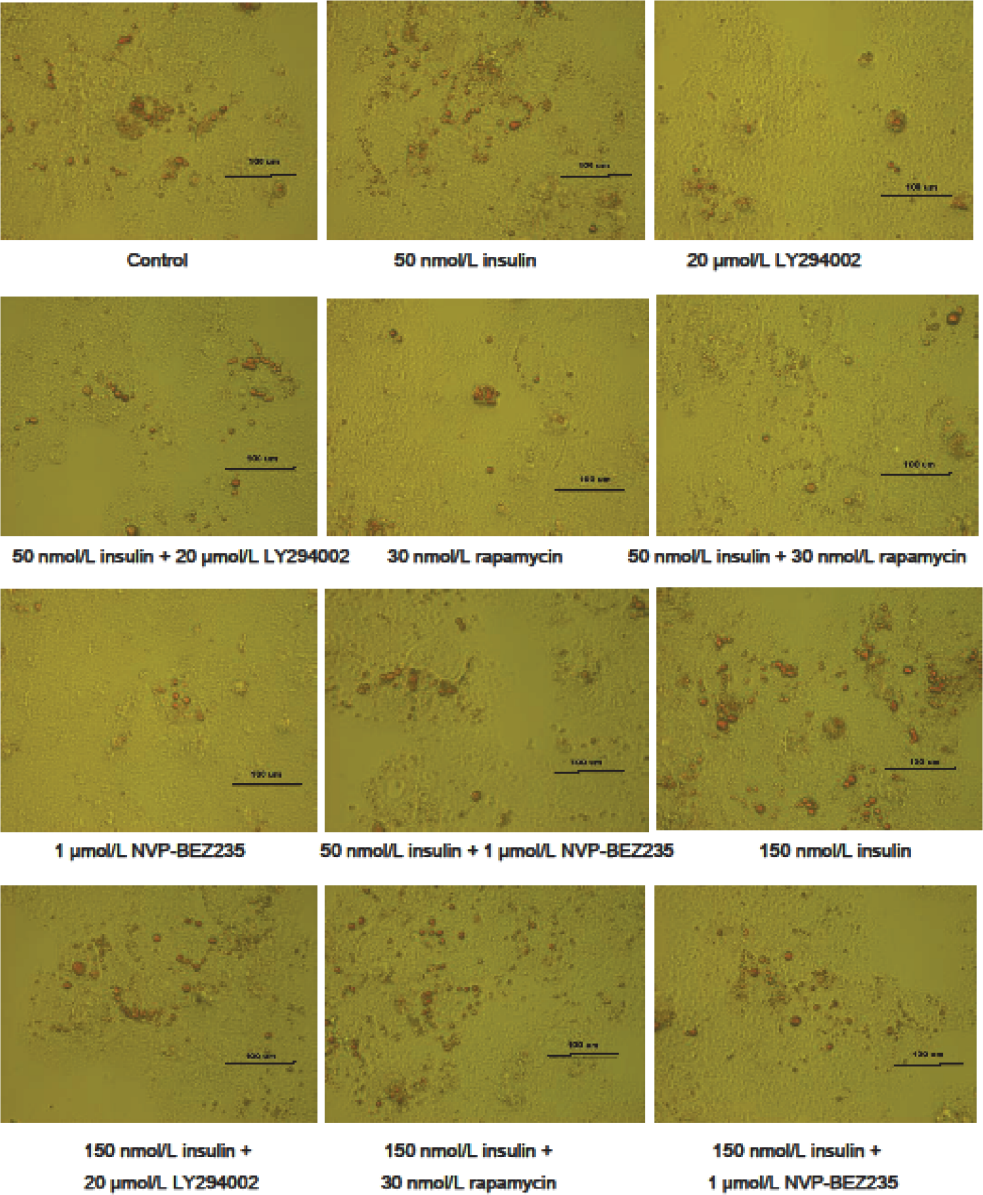
Intracellular lipid accumulation as measured by Oil Red O staining. The cells were examined by phase contrast microscopy at 40× magnification.

To verify that the regulation of lipid accumulation by insulin is connected to the PI3K signaling pathway, lipid accumulation was evaluated in cells treated with insulin and a PI3K signaling inhibitor, LY294002. As shown in Figs 4 and 5, treatment with 20 μmol/L LY294002 diminished the insulin-induced increases in intracellular and extracellular TG levels, lipid contents, mRNA levels of genes involved in lipogenesis (SREBP-1, FAS, ACCα, ChREBP, and LXRα), and protein levels of FAS and ACCα. LY294002 treatment also abrogated the inhibitory effects of insulin on the extracellular VLDL concentration, mRNA levels of genes involved in fatty acid oxidation (PPARα, PPARγ, CPT1, and ACOX1) orVLDL-TG assembly and secretion (FoxO1, MTTP, and ApoB), and CPT1 protein levels. An Oil Red O staining assay further indicated that LY294002 treatment inhibited the lipid accumulation induced by insulin (Fig 6). LY294002 also decreased the insulin-induced stimulation of ACCα protein expression (Fig 3). Combined treatment with 150 nmol/L insulin and LY294002 produced greater changes in the measured indicators than treatment with 50 nmol/L insulin and LY294002. These results indicate that insulin regulates lipid accumulation through the PI3K signaling pathway.

### Rapamycin Blocked the Effect of Insulin on Lipid Accumulation

**Fig 7:**
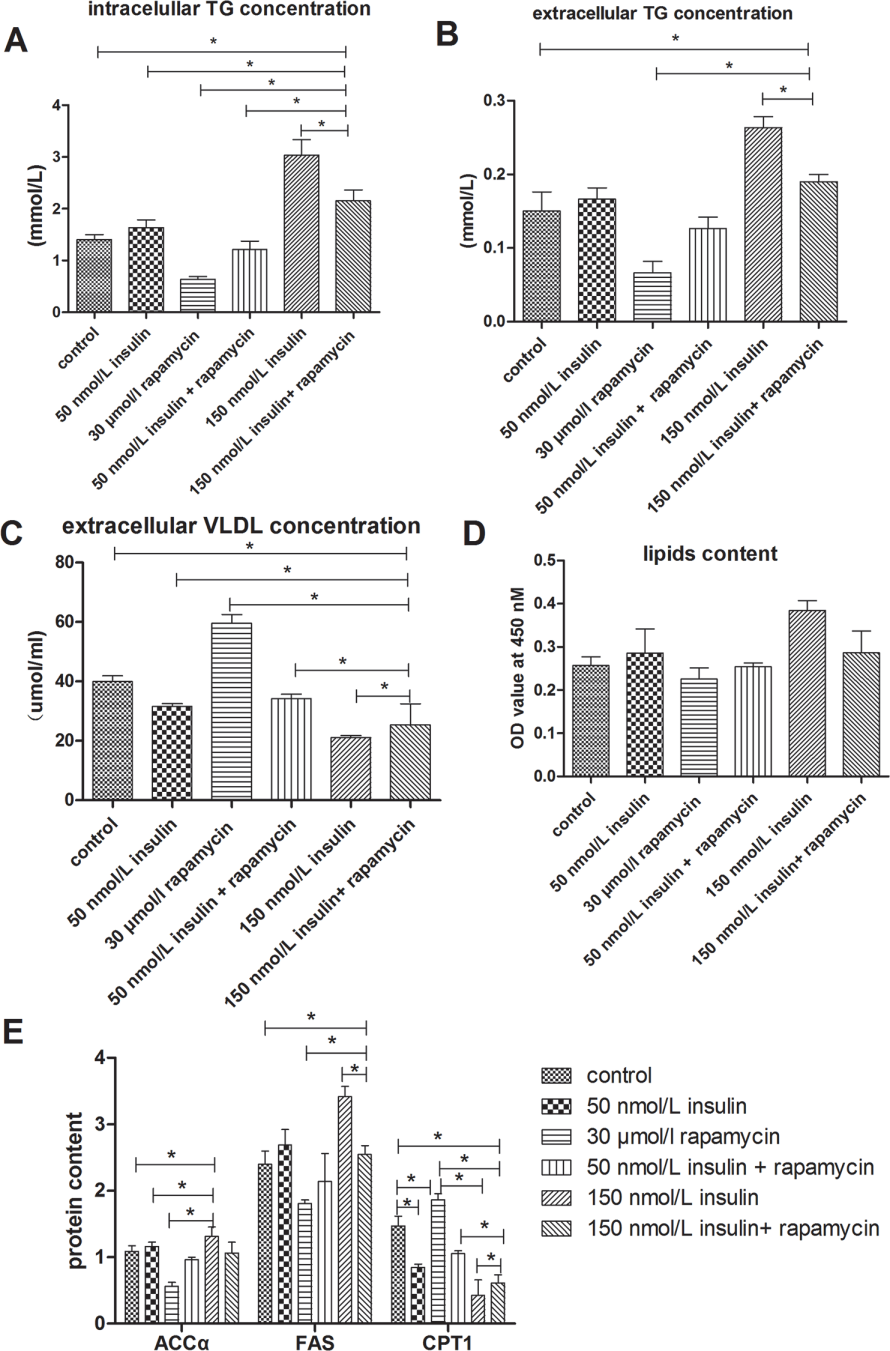
Treatment with rapamycin blocked the effect of insulin on lipid accumulation. **A**, intracellular TG concentrations. **B**, extracellular TG concentrations. **C**, extracellular VLDL concentrations. **D**, Lipid contents were measured by Oil Red O extraction and are shown in optical density value units. **E**, Protein levels of genes involved in lipid metabolism; FAS levels are shown in nmol/ml, whereas ACCα and CPT1 levels are shown in ng/ml. * indicate significant differences among treatments (P <0.05).

**Fig 8:**
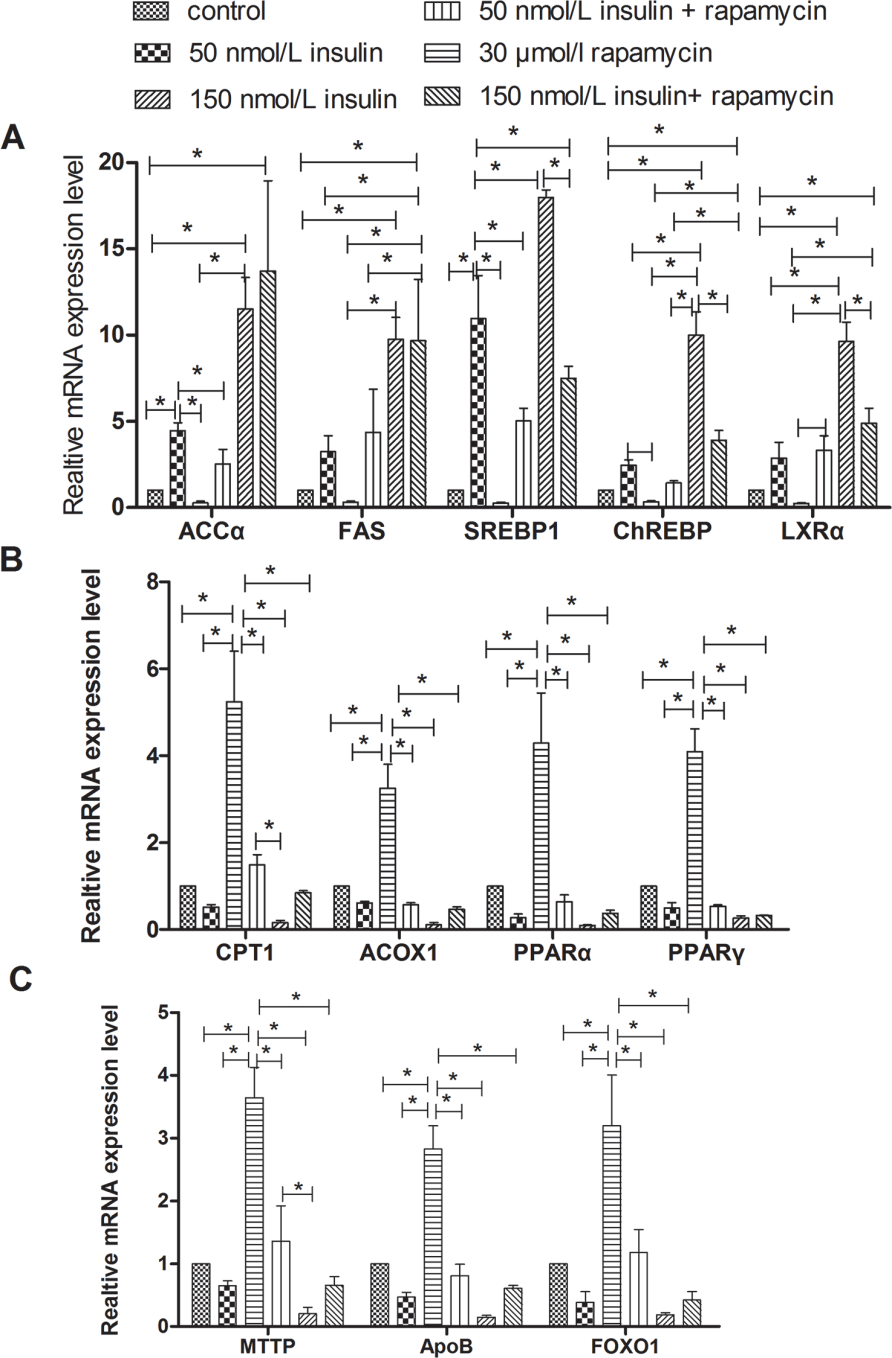
Treatment with rapamycin affected the regulation of insulin on mRNA levels of genes involved in lipid metabolism. **A**, Relative mRNA levels of genes related to lipogenesis. **B**, Relative mRNA levels of genes involved in fatty acid oxidation. **C**, Relative mRNA levels of genes that participate in VLDL-TG assembly and secretion. * indicate significant differences among treatments (P<0.05).

To test the hypothesis that the regulation of lipid accumulation by insulin is connected to the mTOR signaling pathway, lipid accumulation was evaluated in cells treated with insulin and an mTOR signaling inhibitor, rapamycin. As shown in Figs 7 and 8, treatment with 30 nmol/L rapamycin decreased the stimulatory effects of insulin on intracellular and extracellular TG levels, lipid contents, mRNA levels of genes involved in lipogenesis (SREBP-1, FAS, ACCα, ChREBP, and LXRα), and protein levels of FAS and ACCα. In addition, 30 nmol/L rapamycin blocked the inhibitory effects of insulin on the intracellular VLDL concentration, mRNA levels of genes involved in fatty acid oxidation (PPARα, PPARγ, CPT1, and ACOX1) or VLDL-TG assembly and secretion (FoxO1, MTTP, and ApoB), and protein levels of CPT1. An Oil Red O staining analysis further confirmed that 30 nmol/L rapamycin decreased the lipid accumulation induced by insulin (Fig 6). Rapamycin also decreased the insulin-induced up-regulation of ACCα protein expression (Fig 3). Combined treatment with 150 nmol/L insulin and 30 nmol/L rapamycin produced more substantial changes in the measured parameters than treatment with 50 nmol/L insulin plus rapamycin. These results indicate that insulin regulates lipid accumulation through the mTOR signaling pathway.

### NVP-BEZ235 Abrogated the Effect of Insulin on Lipid Accumulation

**Fig 9:**
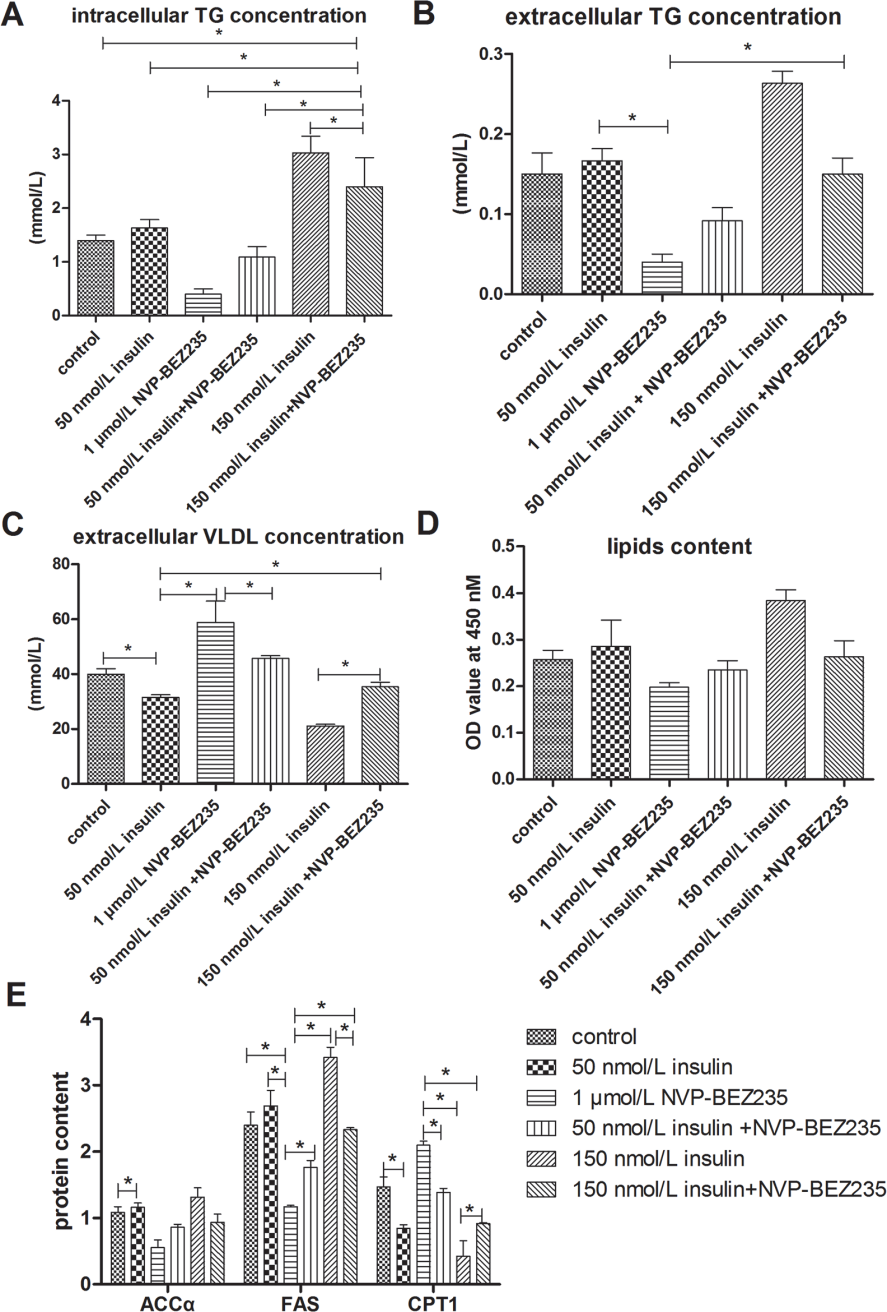
Treatment with NVP-BEZ235 blocked the effect of insulin on lipid accumulation. **A**, intracellular TG concentrations. **B**, extracellular TG concentrations. **C**, extracellular VLDL concentrations. **D**, Lipid contents were measured by Oil Red O extraction and are shown in optical density value units. **E**, Protein levels of genes involved in lipid metabolism; FAS levels are shown in nmol/ml, whereas ACCα and CPT1 levels are shown in ng/ml. * indicate significant differences among treatments (P <0.05).

**Fig 10:**
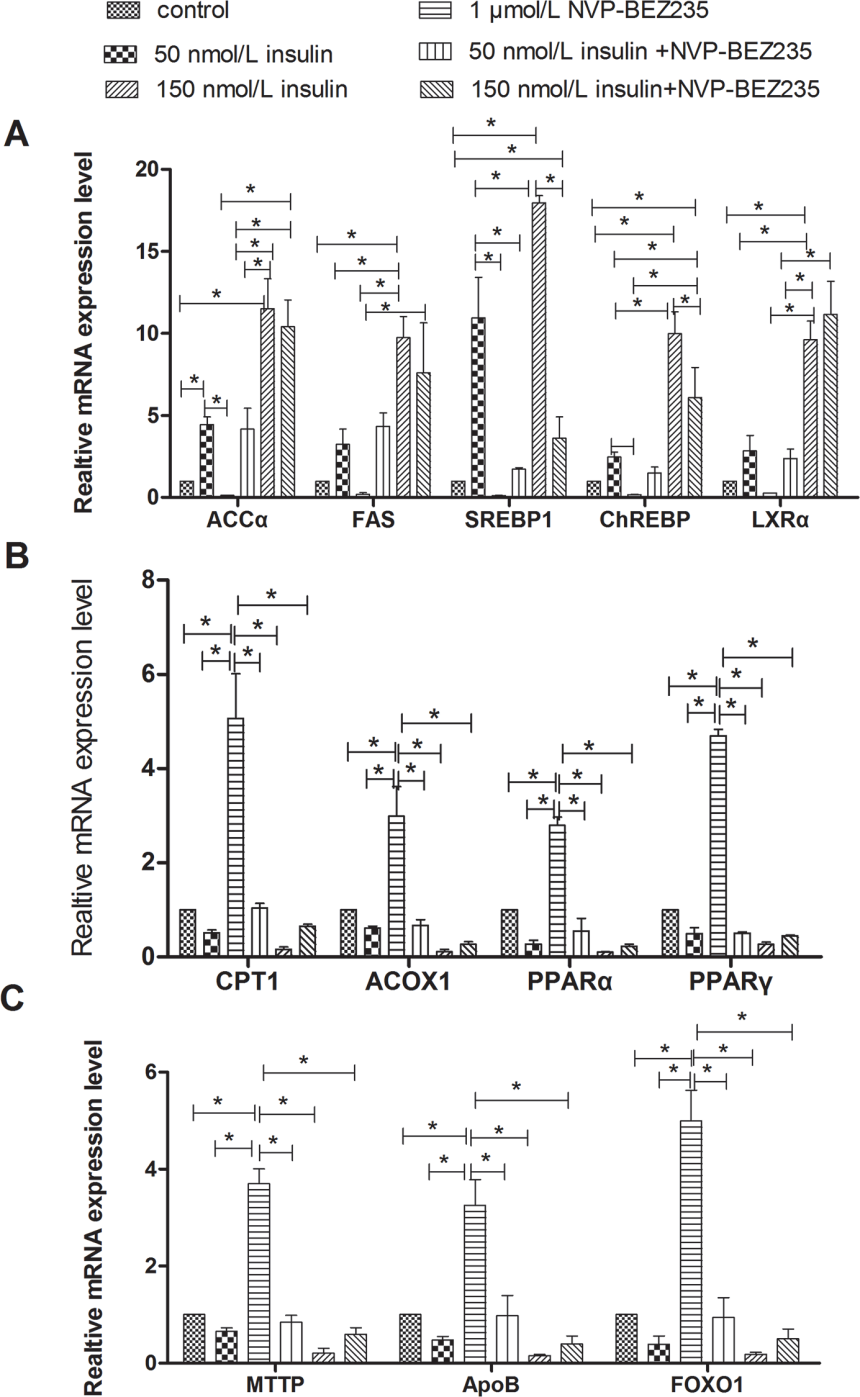
Treatment with NVP-BEZ235 affected the regulation of insulin on mRNA levels of genes involved in lipid metabolism. **A**, Relative mRNA levels of genes related to lipogenesis. **B**, Relative mRNA levels of genes involved in fatty acid oxidation. **C**, Relative mRNA levels of genes that participate in VLDL-TG assembly and secretion. * indicate significant differences among treatments (P<0.05).

To determine whether inhibition of both PI3K and mTOR signaling would have a more intense effect on the regulation of lipid accumulation by insulin, lipid accumulation was evaluated in cells treated with insulin as well as a dual PI3K and mTOR signaling inhibitor, NVP-BEZ235. Similar to treatment with LY294002 and rapamycin, treatment with 1 μmol/L NVP-BEZ235 decreased the stimulatory effects of insulin on intracellular and extracellular TG levels, lipid contents, and mRNA and protein levels of genes involved in lipogenesis (Figs 9 and 10). In addition, treatment with 1 μmol/L NVP-BEZ235 abrogated the inhibitory effects of insulin on intracellular VLDL levels as well as the mRNA and protein levels of genes involved in fatty acid oxidation or VLDL-TG assembly and secretion. Oil Red O staining confirmed that treatment with 1 μmol/L NVP-BEZ235 decreased the insulin-induced lipid accumulation (Fig 6), however, up-regulation of ACCα protein expression by western blot assays was not evident (Fig 3). Combined treatment with insulin and NVP-BEZ235 abrogated the insulin-induced lipid accumulation to a similar degree as treatment with LY294002 and rapamycin, but the changes caused by NVP-BEZ235 were less severe than those caused by LY294002 and rapamycin. These results indicate that insulin regulates lipid accumulation through both the PI3K and mTOR signaling pathways.

## Discussion

To determine whether inhibition of the PI3K-Akt-mTOR pathway altersinsulin-induced hepatic lipid deposition, we used a previously established primary goose hepatocyte model. This system has two advantages. First, by using a single cell type, it is possible to measure the direct effects of the drugs on hepatocytes in the absence of potential secondary effects from other cell types, hormones, or plasma metabolites. Second, measurement of important metabolic fluxes can be performed with relative precision in a culture system. We first tested whether insulin affects lipid deposition in goose primary hepatocytes. The results of these experiments indicate that insulin positively regulates lipid deposition in these cells by activating lipogenesis and inhibiting fatty acid oxidation and VLDL-TG assembly/secretion.

Pharmacological inhibition is a powerful method for studyingthe functions of endogenous proteins in acute hormone action. Moreover, pharmacological inhibition is still by far the most common mechanism of action for drugs in clinical use, demonstrating the importance of developing and characterizing such tools [13].Whether the PI3K-Akt-mTOR pathway is involved in insulin-induced lipid deposition has not been determined. To investigate the role of the PI3K-Akt-mTOR pathway in this process, goose primary hepatocytes were cultured with the chemical inhibitors LY294002, rapamycin, or NVP-BEZ235 for 24h and then with insulin for 24h. Our studies revealed that insulin regulates lipid deposition by activating lipogenesis and inhibiting fatty acid oxidation and VLDL-TG assembly/secretion and that these activities are mediated by the PI3K-Akt-mTOR pathway in primary cultures of goose hepatocytes.

Our results strongly indicate that the PI3K-Akt-mTOR signaling pathway mediates insulin-induced de novo fatty acid synthesis in primary cultures of goose hepatocytes. This result is consistent with the findings of Leavens et al. [14], who showed that genetic ablation of PI3K and Akt in the liver resulted in a marked reduction in insulin-stimulated lipogenesis. A recent study also found that inhibition of PI3K with LY294002 caused a marked reduction in TG levels, regardless of insulin concentration [15]. Other studies have reported that increased or inhibited mTOR activity impairs lipid homeostasis by regulating the lipogenic genes PPARα and PPARγ [16,17]. The role of mTORC1 activation in the metabolic response of the liver to insulin is poorly understood. To verify that insulin induces lipogenesis through the mTOR pathway, we used the mTOR inhibitor rapamycin and the PI3K/mTOR dual inhibitor NVP-BEZ235. The results indicated that inhibition of mTOR by rapamycin or NVP-BEZ235 clearly blocked the insulin-induced increase in lipogenesis. The conclusion that the mTOR pathway is required for insulin-induced lipogenesis in goose primary hepatocytes is consistent with previous findings in mice [18–20], where inhibiting mTORC1 attenuated insulin-mediated induction of the SREBP-1c-dependent lipogenic program and liver steatosis. Our results are also consistent with studies of chicken embryo hepatocytes, where it was shown that rapamycin blocks the insulin-induced increase in SREBP-1c and its target genes [21]. However, there are conflicting observations in the literature regarding the link between the PI3K-Akt-mTOR pathway and lipogenesis in the liver [14]. Some of these discrepancies may be related to the fact that the liver must integrate other signals in addition to insulin. Of particular importance is glucagon, which stimulates adenylyl cyclase to initiate events that oppose the actions of insulin, including the insulin-mediated increase in SREBP-1c mRNA [22].Together with the above studies, our current findings indicate that the PI3K-Akt-mTOR pathway is an essential downstream target of insulin for the proper induction of lipogenesis in goose hepatocytes.

In addition to the lipogenesis pathway, our results indicate that PI3K-Akt-mTOR signaling mediates the insulin-stimulated activation of several major pathways of hepatic fatty acid metabolism, including fatty acid β-oxidation, fatty acid esterification to triglycerides and VLDL-TG secretion in primary cultures of goose hepatocytes. Compared with healthy liver, the activation of insulin signaling triggers a strong induction of the AKT-mTOR cascade that is paralleled by the increased synthesis of fatty acids and triglycerides and a decrease in fatty acid oxidation in rat preneoplastic and neoplastic liver lesions [23]. PI3K and Akt can modulate CPT1 expression to suppress β-oxidation during anabolic growth [24]. Rapamycin-mediated inhibition of mTOR increases the fatty acid oxidation rate in skeletal muscle cells both in vivo and in vitro [25]. Um et al.[26] reported that mice deficient in S6K1, a downstream target of mTOR, increased their in vivo fatty acid oxidation capacity in association with elevated mRNA levels of genes involved in fatty acid oxidation (CPT1 and PPARγ) in skeletal muscle and adipose tissue. The action of CPT1 is the primary regulated step in the mitochondrial oxidation of long-chain fatty acids in hepatocytes under most physiological circumstances [27]. Our studies showed that LY294002, rapamycin, and NVP-BEZ235 all reversed the insulin-induced down-regulation of CPT1 protein and other genes related to fatty acid oxidation (CPT1, ACOX1, and PPARγ). CPT1 is potently inhibited by malonyl-CoA, the first committed intermediate in the opposing pathway of de novo fatty acid biosynthesis. Therefore, when the de novo synthesis pathway is active, elevated levels of malonyl-CoA inhibit CPT1 and prevent the mitochondrial entry and futile oxidation of the newly synthesized fatty acids [28]. It is possible that the increased flux of β-oxidation induced by rapamycin involves decreased production of the physiological CPT1 inhibitor malonyl-CoA. Our results showed that insulin significantly down-regulated the mRNA and protein levels of genes involved in fatty acid oxidation in goose hepatocytes and that inhibition of the PI3K-Akt-mTOR pathway by LY294002, rapamycin, or NVP-BEZ235 blocked the insulin-mediated decreases in the mRNA and protein levels of genes related to fatty acid oxidation. Therefore, the PI3K/Akt/mTOR signaling pathway contributes to the insulin-mediated decrease in fatty acid oxidation in goose hepatocytes and consequently promotes the accumulation of fatty acids in liver cells.

Insulin is one of several metabolic signals that reduce VLDL production. Insulin has been shown to acutely suppress hepatic apoB100-containing lipoprotein particle production both in vitro [29] and in vivo [30,31] in animals and humans. Our results show that insulin decreased intracellular VLDL levels and decreased MTTP protein levels and the mRNA levels of genes involved in VLDL-TG assembly and secretion, indicating that insulin suppresses VLDL assembly and secretion in goose hepatocytes. The mechanism of this action is not clear. It has been reported that insulin diminishes VLDL secretion through several signaling pathways, including the PI3K pathway [32–34]. MTTP is necessary for the assembly of nascent lipoprotein particles, and the strongest inhibitory effect of insulin on VLDL assembly and secretion appears to be at the level of MTTP expression [35]. As FOXO1 plays a role in VLDL-TG assembly by regulating MTTP, and ApoB regulates VLDL synthesis in the liver, the down-regulation of these genes should disrupt VLDL-TG assembly and secretion. Our study showed that inhibition of the PI3K-Akt-mTOR pathway by LY294002, rapamycin, or NVP-BEZ235 released the insulin-induced suppression of intracellular VLDL levels, MTTP protein expression, and mRNA expression of MTTP, ApoB, and FOXO1 in goose hepatocytes. The PI3K-Akt-mTOR signaling pathway may therefore mediate the inhibitory effect of insulin on VLDL assembly and secretion.

In summary, our experiments show, for the first time, that the PI3K-Akt-mTOR signaling pathway plays an important role in insulin-mediated lipid metabolism in goose hepatocytes.
